# Is Potassium Iodide a Specific in Lobar Pneumonia?

**Published:** 1904-05-14

**Authors:** 


### IS POTASSIUM IODIDE A SPECIFIC IN LOBAR PNEUMONIA?

We have now grown pretty well accustomed to hearing of extraordinary successes following therapeutic heroism, and consequently it is without much astonishment that we read of the administration of 3,000 grains of potassium iodide daily to a patient suffering from pneumonia. Such a case is reported by Dr. Altshul[1] in a paper in which he states that observations on 62 cases of pneumonia treated by himself without a death, and a still larger number of instances under the care of his colleagues with results approximately similar, have caused him to question if iodide of potassium is not as much a specific for lobar pneumonia as is quinine for malaria, or iron for anæmia.

Dr. Altshul states that in his own 62 cases he has been particularly impressed by the influence exerted by this drug on the course and character of the disease. Thus in none of the cases was there the least semblance of a crisis, the termination being invariably by lysis. This applies to both the temperature and the general condition of the patient. The temperature, even in cases of apical pneumonia, was never very high, rarely exceeding 103° F., never above 104° F., when the treatment was commenced early; and complications were never met with. The explanation is due, Dr. Altshul believes, to the inhibitory action of the potassium iodide on the growth of the pneumococcus, and the consequent diminution of toxins produced. In spite of the favourable action of the drug, the duration of the illness is not shortened.

The procedure which was adopted, with trifling modifications, in all cases was to administer an initial dose of at least 10 to 15 grains of potassium iodide immediately the diagnosis of pneumonia was established. This dose was increased by five or 10 grains, according to the severity of the case, every two or three hours, day and night, until defervescence ensued. However soft the pulse became the dose was kept up so long as the heart-beats remained regular. The iodide was prescribed in 50 per cent. solution, and was administered in milk. At the commencement of treatment a slight coryza occasionally appeared, but rapidly cleared up when the doses were increased. In concluding his paper, Dr. Altshul urges that the one guide in dosage must be the condition of the heart as shown by the regularity of its beat and the distinctness of the pulmonary second sound. Failure to obtain favourable results is due, the author thinks, to faulty methods and lack of courage, rather than to ineffectiveness of the method which he recommends.

[1] Med. Rec., March 26.

